# 8-oxo-7,8-dihydroguanosine (8-oxo-Guo) drives pulmonary inflammatory pathways through pattern recognition receptors

**DOI:** 10.1016/j.redox.2026.104211

**Published:** 2026-05-14

**Authors:** Rui Li, Jia-Xin Pan, Gang Sheng, Xu-Fan Gao, Ran Huan, Ruo-Mei Qi, Jin Li, Jian-Ping Cai

**Affiliations:** a Department of Basic Innovation Research, Beijing Hospital, National Center for Gerontology, National Clinical Research Center for Gerontology, The Key Laboratory of Geriatrics of NHC, Beijing Key Laboratory of Aging Mechanism and Intervention Research on Aging-Related Diseases, Institute of Geriatric Medicine, Chinese Academy of Medical Sciences & Peking Union Medical College, China; b Beijing Hospital, National Center for Gerontology, National Clinical Research Center for Gerontology, The Key Laboratory of Geriatrics of NHC, Institute of Geriatric Medicine, Chinese Academy of Medical Sciences & Peking Union Medical College, Beijing, China; c Dept of Pulmonary and Critical Care Medicine, Peking University Shougang Hospital, Beijing, China

**Keywords:** 8-Oxo-7,8-dihydroguanosine, Pulmonary inflammation, NOD2 receptor, Single-stranded RNA

## Abstract

8-oxo-7,8-dihydroguanosine (8-oxo-Guo) is a key biomarker of oxidative stress and nucleic acid damage, the levels of which positively correlate with aging and age-related diseases. Notably, in inflammatory diseases, 8-oxo-Guo levels are significantly elevated. *In vivo* mouse experiments showed that a tail vein injection of 8-oxo-Guo increased inflammatory factor expression in peripheral blood and lung tissue and increased the proportion of M1-type macrophages in bronchoalveolar lavage fluid, indicating the induction of macrophage polarization and pulmonary inflammation. *In vitro* studies of mouse alveolar macrophages revealed that 8-oxo-Guo activates inflammatory pathways via Nucleotide-binding oligomerization domain-containing protein 2 (NOD2), Toll-like receptor 2 (TLR2), and NOD-, LRR-, and pyrin domain-containing protein 3 (NLRP3) receptors synergistically with single-stranded RNA (e.g., polyuridylic acid or uracil-rich microRNA). NOD2 appears as a core regulatory target, in which its inhibition effectively reduces inflammation. The present study elucidates a novel mechanism whereby endogenous 8-oxo-Guo drives pulmonary inflammation through these receptors in alveolar macrophages, indicating that 8-oxo-Guo is a key inflammatory initiator in alveolar macrophages and mice.

## Highlights

• 8-oxo-Guo actively drives pulmonary inflammation, beyond being a mere biomarker.
• 8-oxo-Guo induces M1 macrophage polarization and elevates inflammatory factors.
• 8-oxo-Guo synergizes with ssRNA to activate inflammation via the PRRs.
• NOD2 is the core regulator in this synergistic activation by 8-oxo-Guo and ssRNA.
• A novel mechanism that oxidative RNA directly regulates pulmonary inflammation.

## Graphical abstract

**8-oxo-Guo and ssRNA synergistically activate the NOD2-mediated PRR signaling pathway to enhance inflammation.** (Created with Figdraw, ID: AARTY1163b).

**Figure:**
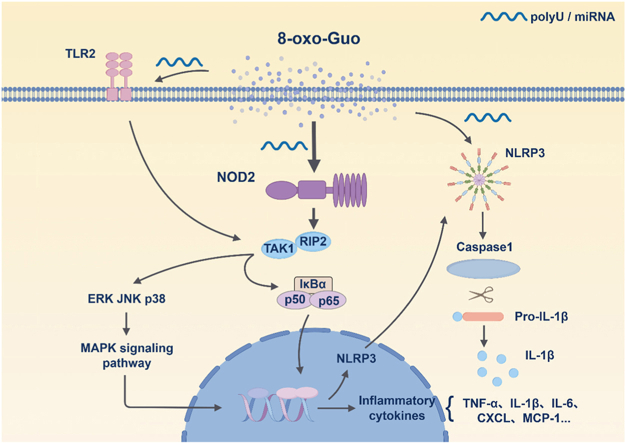

## 1 Introduction

In 1956, Denham Harman introduced the “free radical theory of aging,” which has become one of the most influential theories in the field of gerontology [1]. This theory posits that aging is a process that is driven by the accumulation of damage to cells and their components, such as nucleic acids, proteins, and lipids, that is caused by free radicals that are generated within the body. This cumulative damage ultimately results in the deterioration of tissue function and aging of the organism [2].

Nucleic acids are carriers of genetic information and particularly susceptible to damage during oxidative stress [3]. Compared to DNA, which is protected by its double-helix structure and associated histones, RNA is more vulnerable to oxidative damage because of its single-stranded structure and lack of histone protection [4]. Among nucleobases in RNA, guanine is especially prone to oxidation because of its low oxidation potential, leading to the formation of 8-oxo-7,8-dihydroguanine (8-oxo-G) upon exposure to reactive oxygen species (ROS) [5,6]. Consequently, 8-oxo-G is considered a significant biomarker of oxidative stress and nucleic acid damage [7]. RNA that contains 8-oxo-G in cells or 8-oxoGTP in the nucleotide pool can undergo further degradation into free 8-oxo-7,8-dihydroguanosine (8-oxo-Guo). This metabolite then enters the bloodstream and is subsequently excreted in urine [8].

Our prior research demonstrated that 8-oxo-Guo in urine serves as a robust biomarker of aging [[9], [10], [11], [12]] and is also as a valuable indicator for assessing human health. We examined urinary 8-oxo-Guo levels across various disease states and models, including neurodegenerative disorders [[13], [14], [15]], cardiovascular diseases [[16], [17], [18], [19]], diabetes [[20], [21], [22]], renal diseases [[23], [24], [25], [26]], cancer [27,28], hepatitis [29,30], and enteritis [[31], [32], [33]]. Our findings revealed that 8-oxo-Guo levels are generally elevated and positively correlate with disease progression. This increase is particularly pronounced in inflammatory diseases. For example, patients who were infected with hepatitis B virus and hepatitis C virus had significantly higher urinary 8-oxo-Guo levels compared with healthy controls, with a strong correlation with liver fibrosis [29,30]. Notably, following the onset of bacteria-induced enteritis, there was a significant increase in 8-oxo-Guo levels within RNA chains of such organs as the intestine, liver, spleen, and brain and in urine, which positively correlated with white blood cell counts and various inflammatory cytokines [31]. Consequently, urinary 8-oxo-Guo can be a biomarker for evaluating the severity of infections that are caused by microbial pathogens [32,33].

The lungs are continuously exposed to the external environment and particularly susceptible to invasion by environmental pollutants and respiratory pathogens, which can lead to the development of chronic bronchitis, asthma, and chronic obstructive pulmonary disease [[34], [35], [36]]. Pulmonary macrophages serve as a critical line of defense against pathogens and environmental pollutants [37,38]. Pattern recognition receptors (PRRs) are extensively distributed within pulmonary macrophages [39]. During pulmonary infections, Toll-like receptors (TLRs) on macrophages function as membrane-bound receptors, broadly recognizing viral nucleic acids and bacterial components. This recognition initiates a cascade of proinflammatory reactions, mediating the release of proinflammatory factors, facilitating neutrophil infiltration, and playing a crucial role in the clearance of respiratory pathogens [40,41]. NOD-like receptors (NLRs) act as cytoplasmic sensors that respond to pathogen invasion or tissue damage and are significant contributors to various inflammatory diseases, including both infectious and aseptic (acute and chronic) diseases. The activation of NLRs results in the production and release of proinflammatory factors and may induce cell death [41,42].

Current research indicates that nucleic acid oxidation metabolites are implicated in immune responses. Bacsi et al. [43] reported that 8-oxo-G can induce the activation of dendritic cells that derive from monocyte-derived dendritic cells (moDCs). Additionally, Miyake et al. [44,45] revealed that guanosine (Guo), 2′-deoxyguanosine (dGuo), 8-hydroxyguanosine (8-OHG), and 8-hydroxydeoxyguanosine (8-OHdG) can activate TLR7 in the presence of polyuridylic acid (polyU), thereby triggering an inflammatory response.

The present study primarily involved *in vivo* animal experiments and *in vitro* cell experiments to investigate the role of endogenous 8-oxo-Guo, which is produced through RNA oxidation and 8-oxoGTP metabolism, in the development of pulmonary inflammation by activating inflammatory pathways of mouse alveolar macrophages (AMs). Mechanistically, 8-oxo-Guo synergized with single-stranded RNA (ssRNA), including polyU and uracil-rich microRNA, to activate inflammatory signaling through NOD2, TLR2, and NOD-, LRR-, and pyrin domain-containing protein 3 (NLRP3) in AMs, with NOD2 exhibiting the strongest contribution among the three receptors. Collectively, our findings demonstrate for the first time that 8-oxo-Guo acts as an endogenous inflammatory mediator that drives pulmonary inflammation through pattern recognition receptor-dependent signaling pathways.

## 2 Methods

### 2.1 Cell culture and reagents

Mouse AMs (MH-S cells) and their specific culture medium were obtained from Pricella Biotechnology (Wuhan, China), and mouse alveolar epithelial cells (MLE-12 cells) and their respective medium were obtained from iCellbioscience (Shanghai, China). All cell cultures were maintained in a controlled environment at 37 °C with 5% CO_2_.

### 2.2 Cell co-culture and reagents

MH-S cells were seeded in six-well plates and cultured until they reached 80% confluence. Subsequently, the culture medium was replaced with fresh medium that contained 2 mM 8-oxo-Guo and/or 20 μg/ml polyU, and the cells were stimulated for 24 h. The resulting cell culture supernatant was collected and centrifuged at 3000 × *g* for 5 min to eliminate cellular debris. The supernatant was then sterilized by filtration through a 0.22 μm membrane to produce CM. This CM was combined with fresh MLE-12 complete medium in a 1:1 vol ratio to formulate the treatment medium. For the control group, an identical procedure was followed using supernatant from unstimulated MH-S cells. MLE-12 cells were pre-seeded in culture plates and allowed to adhere overnight. The original medium was subsequently replaced with the designated treatment medium. Following a 24-h incubation period, the cells were harvested for subsequent analysis.

### 2.3 Cell viability assay

MH-S or MLE-12 cells were initially seeded in 96-well plates. Upon cell adherence, the culture medium was substituted with a medium that contained 2 mM 8-oxo-Guo and/or 20 μg/ml polyU or substituted with CM. After a 24-h incubation period, the existing medium was replaced with fresh medium that was supplemented with 10% CCK-8 reagent (NCM Biotech, Suzhou, China), and the cells were incubated at 37 °C for an additional 30 min. Absorbance was subsequently measured at a wavelength of 450 nm using a microplate reader. The viability of MH-S cells was determined using the following formula: *(Optical density value of treatment group/Optical density value of control group) × 100%*.

### 2.4 Detection of apoptosis

MH-S cells were seeded in 24-well plates and exposed to 2 mM 8-oxo-Guo and/or 20 μg/ml polyU in the culture medium for 24 h at 37 °C. The impact of 8-oxo-Guo and polyU on the apoptosis of MH-S cells was evaluated utilizing an Annexin V-FITC/PI Apoptosis Detection Kit (Elabscience, Wuhan, China). A two-step protocol was performed in accordance with the manufacturer's instructions. Post-incubation, the samples were gently resuspended and immediately subjected to flow cytometry analysis. Data analysis was performed using CytExpert.

### 2.5 Western blot

Cells were lysed using Western complete lysis buffer (Beyotime, Shanghai, China), supplemented with 1× protease inhibitor and 1× phosphatase inhibitor (both from Selleck, China). The lysates were centrifuged, and the supernatant was collected to isolate total protein. Protein concentrations were quantified utilizing the Pierce BCA Protein Assay Kit (Thermo Scientific, Waltham, MA, USA). The total proteins were resolved on 4-20% FuturePAGE precast gels and subsequently transferred onto polyvinylidene fluoride membranes. Membranes were blocked with 5% skim milk at room temperature for 1 h. Thereafter, membranes were sectioned according to molecular weights of the target proteins and incubated with appropriate primary antibodies at 4 °C overnight (Supplementary Table 1). Following primary antibody incubation, membranes were washed three times with TBST buffer and then incubated with corresponding horseradish peroxidase-conjugated secondary antibodies (Beyotime, Shanghai, China) at room temperature for 1 h. Protein expression was detected using an ultrasensitive ECL chemiluminescence kit (NCM Biotech, Suzhou, China) on a Tanon 5200 Multi automated chemiluminescence/fluorescence imaging system (Tanon, Shanghai, China).

### 2.6 Cytokine detection

MH-S cells were cultured in 12-well plates and incubated for approximately 24 h until reaching approximately 80% confluence. Subsequently, stimulants were introduced into the culture medium. The ssRNA that was utilized in this study was synthesized by Genema Biotech (Suzhou, China; Supplementary Table 2). The NOD2 inhibitor GSK717 (HY-136555) and NLRP3 inhibitor MCC950 (HY-12815A) were obtained from MedChemExpress (MCE), and the TLR2 inhibitor Cu-CPT22 (S8677) was obtained from Selleck.

Following a 4-h treatment period, the cell culture supernatant was collected for analysis, and cytokine secretion levels were quantified using ELISA. The cytokine concentrations in the supernatant were measured using the following ELISA kits: Mini Sample Mouse TNF-α ELISA Kit (E-MSEL-M0002, Elabscience, Wuhan, China), Mouse Growth Regulated Oncogene β/CXCL2 ELISA Kit (E-MSEL-M0002, Elabscience, Wuhan, China), High Sensitivity Mouse IL-1β ELISA Kit (E-HSEL-M0001, Elabscience, Wuhan, China), and Mini Sample Mouse MCP-1 ELISA Kit (E-MSEL-M0012, Elabscience, Wuhan, China).

### 2.7 RNA extraction and RT-qPCR

Following 2-h stimulation of MH-S cells with 8-oxo-Guo and/or polyU, total RNA was extracted using the SteadyPure Universal RNA Extraction Kit (Accurate Biotechnology, Hunan, China). The extracted RNA was reverse transcribed into complementary DNA (cDNA) using the First-strand cDNA Synthesis Mix (Lablead, Beijing, China). qPCR was performed on an Archimed X4 system (ROCGENE, Beijing, China) by employing 2× Realab Green PCR Fast mixture (Lablead, Beijing, China). The primer sequences are listed in Supplementary Table 3. GAPDH served as the internal reference gene, and relative gene expression levels were quantified by the 2^−ΔΔCt^ method.

### 2.8 RNA sequencing

Total RNA extraction was performed under the following conditions: (*i*) MH-S cells were stimulated with 8-oxo-Guo and/or polyU for 2 h, and (*ii*) MLE-12 cells were treated for 24 h with CM that was derived from MH-S cells that were stimulated for 24 h with 8-oxo-Guo and/or polyU. RNA extraction was performed using the SteadyPure Universal RNA Extraction Kit (Accurate Biology, Hunan, China). The purity and concentration of RNA were evaluated using a NanoDrop 2000 spectrophotometer (Thermo Scientific, USA), and RNA integrity was assessed with an Agilent 2100 Bioanalyzer (Agilent Technologies, Santa Clara, CA, USA). Transcriptome libraries were constructed using the VAHTS Universal V5 RNA-seq Library Prep Kit in accordance with the manufacturer's instructions. The transcriptome sequencing and analysis were conducted by OE Biotech (Shanghai, China).

The libraries were sequenced on the Illumina NovaSeq 6000 platform to generate 150-bp paired-end reads. Raw FASTQ reads were processed using fastp to remove low-quality reads and obtain clean reads for downstream analyses. Clean reads were mapped to the mouse reference genome using HISAT2, and gene expression levels were calculated as fragments per kilobase of transcript per million mapped reads (FPKM). Gene read counts were generated using HTSeq-count. Principal component analysis (PCA) and data visualization were performed in R (v3.2.0) to evaluate biological reproducibility among samples. Differentially expressed genes (DEGs) were identified using DESeq2, with genes meeting the criteria of q value < 0.05 and fold change >2 or < 0.5 defined as significant DEGs. GO, KEGG pathway, and WikiPathways enrichment analyses were performed based on the hypergeometric distribution algorithm to identify significantly enriched functional categories. Gene set enrichment analysis (GSEA) was conducted using predefined gene sets ranked according to differential expression between groups. All visualization plots were generated using the OECloud platform (https://cloud.oebiotech.cn).

### 2.9 Molecular docking

*Modeling mmu-miR-23a-3p structure.* The three-dimensional structure of mmu-miR-23a-3p was predicted utilizing the AlphaFold server. From the five models that were generated, the model that had the highest global predicted Local Distance Difference Test (pLDDT) confidence score was selected for further analysis. The pLDDT score, which ranges from 0 to 100, serves as an indicator of prediction reliability for each residue, with higher scores indicating higher reliability.

*Docking 8-oxo-Guo with NOD2.* The molecular docking of 8-oxo-Guo with NOD2 was performed using the CB-Dock2 web server. The two-dimensional structure file of 8-oxo-Guo was sourced from the PubChem database, and the protein structure of NOD2 (AF-ID: Q8K3Z0) was retrieved from the AlphaFold database. The platform automatically incorporated hydrogen atoms and assigned charges. Binding affinity, expressed as kcal/mol, was computed, and the conformation with the lowest energy was selected as the optimal docking model. Molecular interactions were analyzed and visualized using Protein-Ligand Interaction Profiler and PyMol 2.6 software.

*Docking mmu-miR-23a-3p with NOD2.* Protein-RNA docking was performed using the HDOCK server, with the model exhibiting the most favorable docking score selected for subsequent analysis. The docking score, determined by the ITScorePP or ITScorePR scoring function, inversely correlates with binding affinity. More negative values indicate a higher likelihood of binding and stronger interactions. An empirical confidence score was computed to assess the binding likelihood: scores greater than 0.7 denote high probability, scores between 0.5 and 0.7 suggest a potential possibility, and scores lower than 0.5 suggest a low likelihood. Hydrogen bonding interactions and critical amino acid residues at the binding interface were examined using PyMol 2.6 software.

### 2.10 In vivo mouse experiments

Male C57BL/6 N mice, 10-12 weeks old, were obtained from SPF (Beijing) Biotechnology. Guo or 8-oxo-Guo, dissolved in 1× phosphate-buffered saline (PBS) that contained 10% mouse serum, was administered via a tail vein injection at 4 μg/kg body weight. Control mice received an equivalent volume of vehicle (1× PBS with 10% mouse serum). After 4 h, plasma was collected from control, Guo-treated, and 8-oxo-Guo-treated mice (*n* = 5/group). Concentrations of TNF-α, MIP-2, and MCP-1 were quantified using ELISA.

Four hours after the injection, both control and 8-oxo-Guo-treated mice were euthanized via gentle cervical dislocation. Bronchoalveolar lavage fluid was subsequently collected using 1× PBS. For analysis, BALF cells from every two mice were pooled to form a single sample. To block Fc receptors, the cells were incubated with purified anti-mouse CD16/32 antibody (catalog no. 101302, BioLegend, San Diego, CA, USA; 3 μl/tube) on ice for 15 min. The cell suspensions were then stained with the following fluorescently labeled antibodies for 20 min on ice while shielded from light: APC/Cyanine7 Anti-Mouse CD45 (catalog no. 103115, BioLegend), PE-Cy 7 Hamster Anti-Mouse CD11c (catalog no. 561022, BD Pharmingen, Franklin Lakes, NJ, USA), APC Anti-Mouse F4/80 (catalog no. 123115, BioLegend), and BB700 Rat Anti-Mouse CD86 (catalog no. 742120, BD OptiBuild, Franklin Lakes, NJ, USA). Following fixation and permeabilization, intracellular staining was conducted using FITC Anti-Mouse CD206 (MMR) Antibody (catalog no. 141703, BioLegend) at room temperature for 20 min in the dark. The cells were washed twice and subsequently analyzed by flow cytometry.

In a subsequent experimental setup, a co-stimulation group that received both 8-oxo-Guo and mmu-miR-23a-3p was formed. A mixture of 8-oxo-Guo (4 μg/kg) and mmu-miR-23a-3p (0.33 mg/kg) was administered via the tail vein. Plasma samples were collected after 4 h, and concentrations of 200 cytokines were quantified using a RayBio QAM-CAA-4000 array (RayBiotech, Norcross, GA, USA). Following systemic perfusion through the heart, lung tissues were harvested from control mice, mice that were treated with 8-oxo-Guo, and mice that were co-treated with 8-oxo-Guo and mmu-miR-23a-3p 4 h post-injection. Lung homogenates were prepared in 1× PBS and centrifuged to obtain supernatants. The concentrations of CXCL1, CCL2, CCL3, CCL4, CCL5, GM-CSF, IL-1β, IL-2, IL-4, IL-6, IL-10, IL-12p40, IL-12p70, TNF-α, and IFN-γ in the supernatants were measured using the ABplex Mouse Cytokine 15-Plex Assay Kit (catalog no. RK05203, Abclonal, Wuhan, China).

All animal experiments were conducted in strict accordance with the Guidelines for the Management of Laboratory Animals. The experimental protocol received approval from the Animal Ethics Committee of Beijing Shenrui Biotechnology (approval no. SR20241114), ensuring full compliance with ethical standards.

### 2.11 Data analysis

Quantitative data are expressed as the mean ± standard deviation. Differences between two groups were assessed using the unpaired Student's *t*-test, and comparisons among multiple groups were conducted via one-way analysis of variance. All statistical analyses were performed using GraphPad Prism 8.0.1 software. Values of *p* < 0.05 was considered statistically significant.

## 3 Results

### 3.1 8-oxo-Guo induces M1 polarization in mouse AMs

**Fig. 1:**
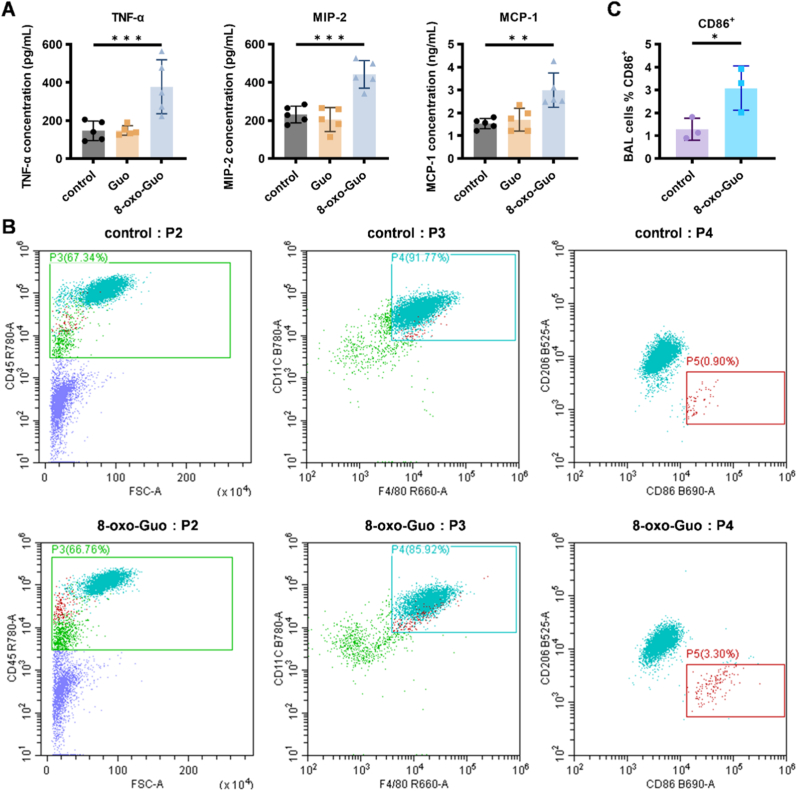
**8-oxo-Guo induces inflammatory response and promotes the M1 polarization of AMs in mice. (A)** Plasma levels of TNF-α, MIP-2, and MCP-1 in mice 4 h after an injection of 8-oxo-Guo or Guo compared with the control group. ∗∗*p* < 0.01, ∗∗∗*p* < 0.001. **(B, C)** Percentages of CD86^+^CD206^-^ cells among CD45^+^F4/80^+^CD11c^+^ AMs in bronchoalveolar lavage fluid from mice that were injected with 8-oxo-Guo compared with the control group. ∗*p* < 0.05.

To investigate the proinflammatory potential of 8-oxo-Guo, mice were administered with either 8-oxo-Guo or Guo via a tail vein injection. After 4 h, plasma levels of proinflammatory cytokines were quantified. Compared to the control group, 8-oxo-Guo significantly elevated concentrations of inflammatory cytokines and chemokines, including tumor necrosis factor-α (TNF-α), macrophage inflammatory protein-2 (MIP-2), and monocyte chemoattractant protein-1 (MCP-1). Conversely, Guo did not elicit similar increases (Fig. 1A).

A detailed flow cytometric analysis of bronchoalveolar lavage fluid (BALF) identified a population of CD45^+^CD11c^+^ macrophages, with approximately 90% exhibiting F4/80^+^CD11c^+^ double-positive markers. This suggests that these cells were predominantly resident AMs rather than monocyte-derived macrophages (Mo-Mφ) that were recruited from bone marrow. Subsequent experiments focused on the expression of CD86 and CD206 in these resident AMs. Compared with the control group, the 8-oxo-Guo-treated group exhibited a 2.4-fold increase in the proportion of CD86^+^CD206^-^ AMs, accompanied by an increase in CD86 fluorescence intensity (Fig. 1B and C). Given that CD86 is a well-established marker of M1-type macrophages, these findings imply that 8-oxo-Guo promotes the polarization of AMs toward the M1 phenotype. M1 macrophages are characteristically proinflammatory and have the potential to intensify pulmonary inflammatory responses. Therefore, the polarization of macrophages that is induced by 8-oxo-Guo may have a substantial impact on the pulmonary immune microenvironment and inflammatory processes.

### 3.2 8-oxo-Guo and polyU synergistically enhance the inflammatory response in macrophages

Subsequent *in vitro* experiments were conducted to further elucidate proinflammatory effects of 8-oxo-Guo on mouse AMs (MH-S cells). Treatment with escalating concentrations of 8-oxo-Guo over 4 h increased levels of TNF-α and MIP-2 in the culture supernatant as quantified by enzyme-linked immunosorbent assay (ELISA), with statistically significant elevations at concentrations ≥0.5 mM. Conversely, concentrations of interleukin-1β (IL-1β) and MCP-1 remained largely unaltered (Supplementary Fig. 1A).

Previous research demonstrated that guanosine and its derivatives, including Guo, dGuo, 8-OHG, and 8-OHdG, can activate TLR7 in the presence of ssRNA, with uridine-rich sequences showing particularly strong synergistic effects [44,45]. TLR7 and TLR8 are pivotal receptors for ssRNA recognition and play critical roles in immune activation upon viral infection or specific RNA ligand binding [46]. Polyuridylic acid is frequently employed as a representative compound for ssRNA in studies of TLR modulation and RNA-mediated immune regulation [47].

**Fig. 2:**
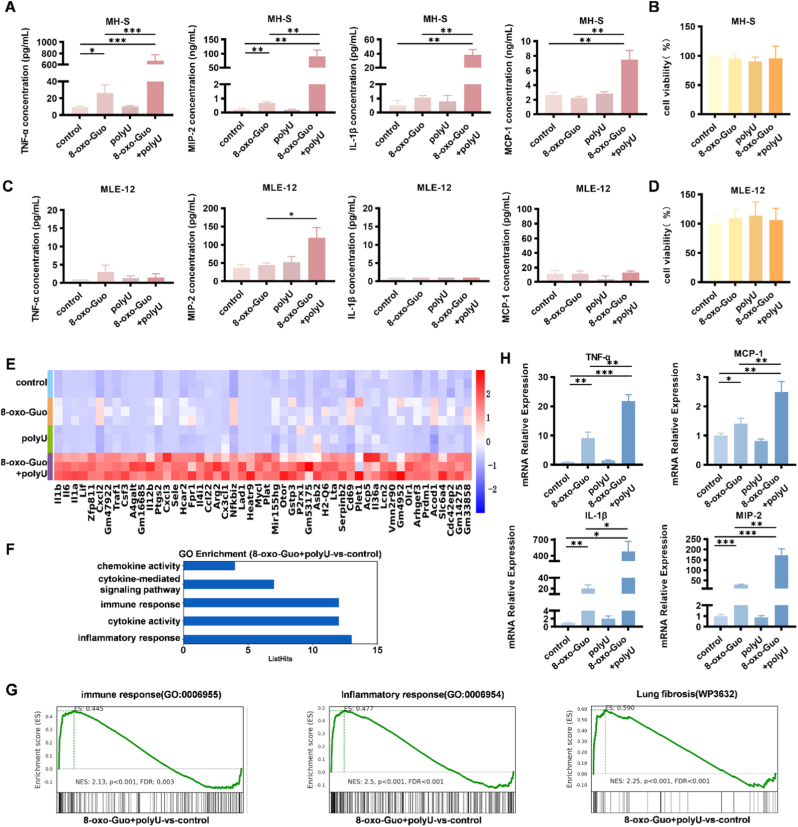
**8-oxo-Guo and polyU synergistically enhanced inflammatory response in MH-S cells. (A)** Concentrations of TNF-α, MIP-2, IL-1β, and MCP-1 in the culture supernatant of MH-S cells following stimulation with 1 mM 8-oxo-Guo and 20 μg/ml polyU. ∗*p* < 0.05, ∗∗*p* < 0.01, ∗∗∗*p* < 0.001. **(B)** Viability of MH-S cells after 24-h stimulation with 1 mM 8-oxo-Guo and 20 μg/ml polyU. **(C)** Concentrations of TNF-α, MIP-2, IL-1β, and MCP-1 in the culture supernatant of MLE-12 cells after 4-h co-stimulation with 1 mM 8-oxo-Guo and 20 μg/ml polyU. ∗*p* < 0.05. **(D)** Viability of MLE-12 cells after 24-h stimulation with 1 mM 8-oxo-Guo and 20 μg/ml polyU. **(E-G)** Transcriptional changes in MH-S cells 2 h after stimulation with 8-oxo-Guo alone or combined with polyU. **(E)** Heatmap of the top 50 significantly upregulated genes following co-stimulation. **(F)** Bar graph of GO enrichment analysis for the top 50 upregulated genes. **(G)** GSEA of DEGs after co-stimulation. **(H)** Relative mRNA expression levels of TNF-α, MIP-2, IL-1β, and MCP-1 in cells after stimulation. ∗∗*p* < 0.01, ∗∗∗*p* < 0.001.

To investigate the potential synergistic proinflammatory effect of 8-oxo-Guo and ssRNA, we co-treated cells with 8-oxo-Guo and polyU. Based on the study by Shibata et al. [44], we defined the optimal treatment conditions for stimulating inflammatory factor secretion in MH-S cells as 1 mM 8-oxo-Guo and 20 μg/ml polyU. Following 4 h of co-treatment, levels of TNF-α, IL-1β, MIP-2, and MCP-1 were markedly elevated compared with 8-oxo-Guo treatment alone, in which TNF-α increased approximately 26-fold, IL-1β increased approximately 37-fold, MIP-2 increased approximately 134-fold, and MCP-1 increased approximately 3-fold (Fig. 2A). Importantly, 24-h co-stimulation did not compromise MH-S cell viability (Fig. 2B) and did not induce apoptosis (Supplementary Fig. 1B).

We next applied the same treatment conditions that were used for MH-S cells to mouse alveolar epithelial cells (MLE-12 cells). The stimulation of MLE-12 cells with 1 mM 8-oxo-Guo and/or 20 μg/ml polyU for 4 h did not induce the secretion of TNF-α, IL-1β, or MCP-1. MIP-2 was detected upon co-stimulation, but its concentration (∼120 pg/ml) was strikingly lower than that secreted by MH-S cells under the same conditions (∼85 ng/ml; Fig. 2C). Moreover, 24-h co-stimulation had no effect on MLE-12 cell viability (Fig. 2D). Our findings indicate that AMs, rather than epithelial cells, exhibit a more pronounced inflammatory response to 8-oxo-Guo stimulation, suggesting that AMs may serve as key effector cells in this process.

To further validate the synergistic effect, we tested a range of concentrations of 8-oxo-Guo and polyU. After 4 h, the secretion of TNF-α, IL-1β, MIP-2, and MCP-1 concentration-dependently increased. A significant response was triggered even at 1 mM 8-oxo-Guo combined with 5 μg/ml polyU (Supplementary Fig. 1C–D). These findings suggest that 8-oxo-Guo and polyU synergistically and concentration-dependently induce inflammation in MH-S cells. Accordingly, 1 mM 8-oxo-Guo and 5 μg/ml polyU were selected as the definitive treatment conditions for this study.

### 3.3 8-oxo-Guo and polyU activate inflammatory responses in AMs via NLRs and TLRs

To comprehensively investigate proinflammatory mechanisms of 8-oxo-Guo, MH-S cells were stimulated with 8-oxo-Guo and polyU for 2 h, followed by transcriptome sequencing (RNA sequencing [RNA-seq]). Analysis of the transcriptome data revealed that the top 50 differentially expressed genes (DEGs) were significantly upregulated in the co-stimulation groups of 8-oxo-Guo and polyU compared with the control group (Fig. 2E). These genes were significantly enriched in pathways that are related to the inflammatory response, cytokine activity, the immune response, cytokine-mediated signal transduction, and chemokine activity (Fig. 2F). Additionally, GSEA indicated that synergistic stimulation by 8-oxo-Guo and polyU was strongly associated with immune responses, inflammatory responses, and lung fibrosis (Fig. 2G). The changes in mRNA levels of TNF-α, MIP-2, IL-1β, and MCP-1 further corroborated the transcriptome sequencing results, demonstrating that co-stimulation with 8-oxo-Guo and polyU led to a more pronounced increase in cellular inflammation compared with stimulation with 8-oxo-Guo alone (Fig. 2H).

**Fig. 3:**
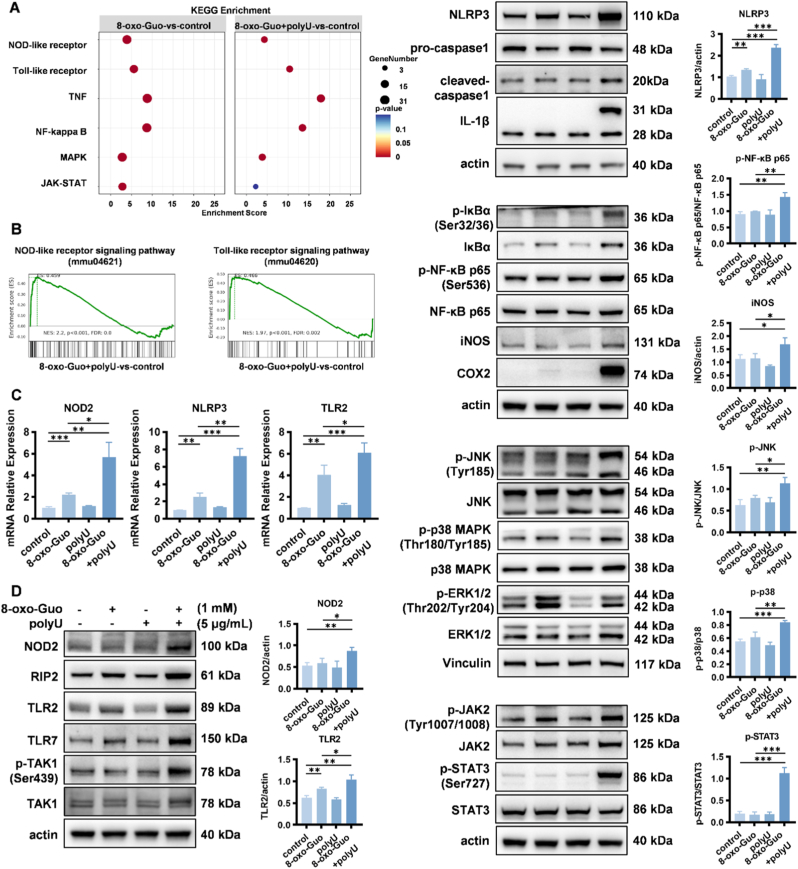
**8-oxo-Guo and polyU stimulate inflammation via NLR and TLR signaling pathways. (A, B)** Transcriptome analysis of MH-S cells following 2-h stimulation with 8-oxo-Guo and/or polyU. **(A)** KEGG pathway enrichment analysis. **(B)** GSEA. **(C)** Relative mRNA expression levels of *NOD2*, *TLR2*, and *NLRP3* in cells after 2-h stimulation with 8-oxo-Guo and/or polyU. ∗*p* < 0.05, ∗∗*p* < 0.01, ∗∗∗*p* < 0.001. **(D)** Protein expression changes and grayscale quantification of key components of NLR, TLR, MAPK, JAK-STAT, and NF-κB signaling pathways following 2-h stimulation with 8-oxo-Guo and/or polyU. ∗*p* < 0.05, ∗∗*p* < 0.01, ∗∗∗*p* < 0.001.

To elucidate the inflammatory signaling pathway that is activated by 8-oxo-Guo, we performed Kyoto Encyclopedia of Genes and Genomes (KEGG) and GSEA enrichment analyses of the transcriptomic data. The KEGG enrichment analysis revealed the significant activation of NLR, TLR, TNF-α, nuclear factor κB (NF-κB), mitogen-activated protein kinase (MAPK), and Janus kinase–signal transducer and activator of transcription (JAK-STAT) signaling pathways in the co-stimulation group (Fig. 3A). The GSEA confirmed strong associations with NLR and TLR pathways (Fig. 3B). Real-time quantitative polymerase chain reaction (RT-qPCR) corroborated the upregulation of NOD2, NLRP3, and TLR2 expression (Fig. 3C). These findings indicate that polyU co-stimulation significantly enhanced 8-oxo-Guo-activated inflammatory signaling.

The Western blot analysis corroborated that in the 8-oxo-Guo stimulation group and polyU group, the combined stimulation of 8-oxo-Guo and polyU resulted in the significant upregulation of key protein expression levels across multiple inflammatory pathways compared with the untreated control group. These pathways included NOD2, receptor interacting protein 2 (RIP2), phosphorylated transforming growth factor-β-activated kinase 1 (p-TAK1), NLRP3, cleaved-caspase-1, and IL-1β within the NLR pathway; TLR2 and TLR7 within the TLR pathway; phosphorylated extracellular signal-regulated kinase (p-ERK), phosphorylated c-Jun N-terminal kinase (p-JNK), and p-p38 within the MAPK pathway; p-JAK and p-STAT3 within the JAK-STAT3 pathway; and phosphorylated inhibitor of κBα (p-IκBα), p-NF-κB p65, inducible nitric oxide synthase (iNOS), and cyclooxygenase 2 (COX2) within the NF-κB pathway (Fig. 3D). Under the aforementioned stimulation conditions, protein expression levels of NOD1, TLR1, TLR3, TLR4, TLR5, TLR6, TLR8, TLR9, and TLR10 did not significantly increase (Supplementary Fig. 2A). The Western blot analysis of MH-S cells revealed a relatively low expression level of TLR7 protein. Consequently, subsequent experiments primarily concentrated on signaling pathways that are associated with the activation of NOD2, NLRP3, and TLR2.

The concentration-dependent synergistic effect between 8-oxo-Guo and polyU was further confirmed at the protein level, indicated by a progressive increase in key inflammatory mediators, including NOD2, RIP2, NLRP3, TLR2, p-TAK1, p-NF-κB p65, and IL-1β, with 1 mM 8-oxo-Guo and escalating polyU concentrations (Supplementary Fig. 2B), consistent with the previous ELISA results.

To further elucidate the dynamics of the inflammatory signaling pathway that was activated by 8-oxo-Guo, we collected cellular proteins and culture supernatants after the administration of 8-oxo-Guo and polyU over various time intervals. Western blot analysis revealed that protein expression levels of NOD2, NLRP3, TLR2, p-NF-κB p65, and IL-1β were upregulated with prolonged stimulation, with pathway activation that was detectable as early as 1 h post-stimulation (Supplementary Fig. 2C). Additionally, concentrations of TNF-α, IL-1β, MIP-2, and MCP-1 in the cell culture supernatant, measured by ELISA, also began to significantly increase within 1 h (Supplementary Fig. 2D). These findings suggest that 8-oxo-Guo and polyU may activate multiple signaling pathways or elevate levels of various inflammatory factors, thereby stimulating additional inflammatory signaling pathways and resulting in an inflammatory amplification effect. Under identical stimulation conditions, MLE-12 cells were exposed for 4 h, but no activation of the aforementioned NOD, TLR, or other signaling pathways was detected by Western Blot (Supplementary Fig. 2E).

### 3.4 Inhibition of NOD2 expression significantly attenuated the inflammatory response induced by 8-oxo-Guo and polyU

Based on these observations, we conclude that 8-oxo-Guo and polyU can significantly activate the NOD2, TLR2, and NLRP3 signaling pathways. We further investigated whether any of these pathways play a predominant role. For this purpose, we employed three receptor-specific inhibitors—the NOD2 inhibitor GSK717, TLR2 inhibitor Cu-CPT22, and NLRP3 inhibitor MCC950—to investigate inflammatory alterations that are induced by 8-oxo-Guo and polyU following the inhibition of NOD2, TLR2, and NLRP3 expression.

**Fig. 4:**
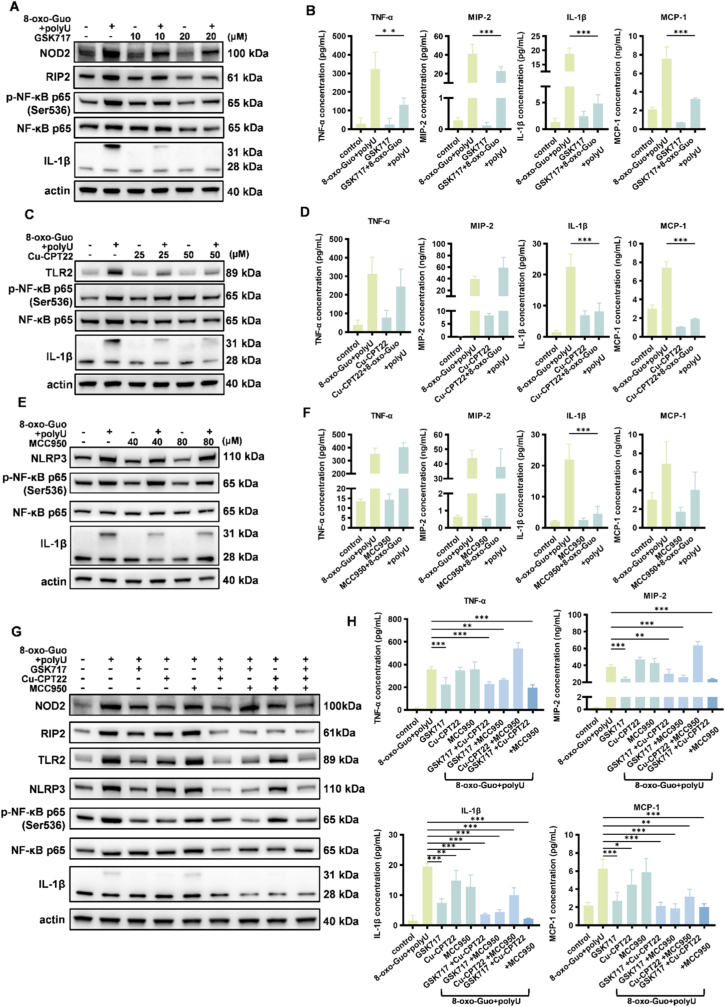
**Dose-dependent suppression of 8-oxo-Guo/polyU-induced inflammation upon NOD2, TLR2, and NLRP3 inhibition. (A-F)** Protein expression changes in NLR, TLR, and downstream signaling pathways (left panels) and concentrations of TNF-α, MIP-2, IL-1β, and MCP-1 in the culture supernatant (right panels) of MH-S cells following 4-h co-stimulation with 8-oxo-Guo and polyU in the presence of GSK717 **(A, B)**, Cu-CPT22 **(C, D)**, or MCC950 **(E, F)**. ∗∗*p* < 0.01, ∗∗∗*p* < 0.001. **(G, H)** Protein expression profiles of relevant signaling pathways **(G)** and cytokine concentrations in the culture supernatant **(H)** of MH-S cells that were treated for 4 h with 8-oxo-Guo and polyU combined with GSK717, Cu-CPT22, or MCC950, either individually or combined. ∗*p* < 0.05, ∗∗*p* < 0.01, ∗∗∗*p* < 0.001.

Initially, we assessed the reduction of protein expression levels of NOD2, TLR2, and NLRP3 at various inhibitor concentrations to identify optimal concentrations for effective inhibition: 10 and 20 μM for GSK717, 25 and 50 μM for Cu-CPT22, and 40 and 80 μM for MCC950 (Supplementary Fig. 3A). Subsequently, these inhibitors were concurrently introduced into the cell culture medium alongside 8-oxo-Guo and polyU. After a 4-h incubation period, we evaluated activation of the signaling pathway and alterations of proinflammatory factor secretion. Western blot and ELISA analyses revealed that GSK717 significantly attenuated the inflammatory response that was elicited by 8-oxo-Guo and polyU, indicated by lower protein expression levels of NOD2, p-NF-κB p65, and IL-1β. Concentrations of the inflammatory factors TNF-α and IL-1β and chemokines MIP-2 and MCP-1 in the cell culture supernatant significantly decreased (Fig. 4A and B, Supplementary Fig. 3B). However, the administration of Cu-CPT22 and MCC950 did not result in a reduction of the inflammatory response as pronounced as with GSK717. Cu-CPT22 inhibited the expression of TLR2 and IL-1β, but protein levels of phosphorylated NF-κB p65 (p-NF-κB p65) continued to rise. While concentrations of IL-1β and MCP-1 in the cell culture supernatant significantly decreased, levels of TNF-α and MIP-2 remained elevated (Fig. 4C and D, Supplementary Fig. 3C). MCC950 effectively reduced NLRP3 protein levels, but following stimulation with 8-oxo-Guo and polyU, protein levels of NLRP3 and p-NF-κB p65 persisted at high levels. Although the concentration of IL-1β in the cell culture supernatant significantly decreased, MCP-1 levels did not notably decrease, and TNF-α and MIP-2 levels continued to increase (Fig. 4E and F, Supplementary Fig. 3D). Furthermore, combined application of the three inhibitors resulted in a more pronounced anti-inflammatory effect, particularly in the presence of GSK717. The most substantial suppression of inflammation was observed when all three inhibitors were administered concurrently (Fig. 4G and H, Supplementary Fig. 3E).

### 3.5 miRNAs with three consecutive U residues synergistically enhance the inflammatory response induced by 8-oxo-Guo

During the processing of viral ssRNA within host cells, the 3’ end of the RNA can undergo extensive uridylation modifications, resulting in the formation of short polyuridylate fragments [48]. Additionally, research by Baker et al. [49] demonstrated that polyU tracts are conservatively present in genomes of coronaviruses and can be specifically cleaved by endonucleases. We found that synthetic polyU can synergistically interact with 8-oxo-Guo to activate inflammatory pathways, suggesting that during viral infection, 8-oxo-Guo may provoke pronounced inflammatory responses in the presence of viral ssRNA. Importantly, under normal physiological conditions, consecutive uridine stretches are naturally found in endogenous microRNAs (miRNAs) [50]. Building on these findings, the present study further investigated whether miRNAs that contain consecutive U sequences can synergistically enhance inflammation induced by 8-oxo-Guo, even in the absence of viral infection.

**Fig. 5:**
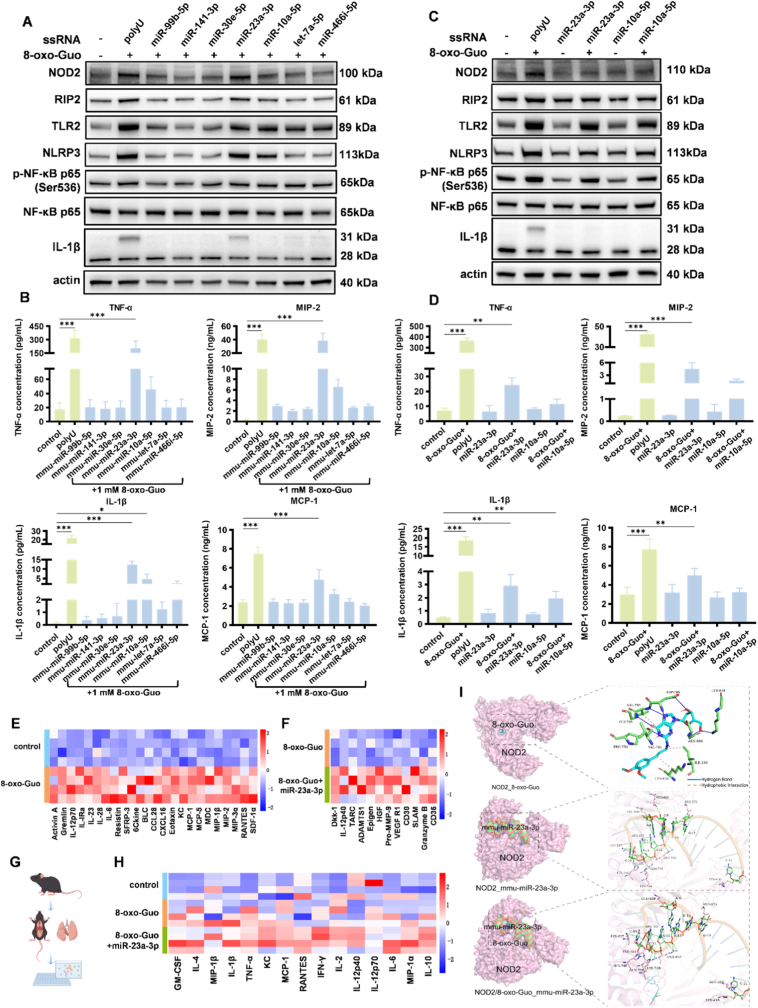
**MicroRNAs that contain three consecutive uridines exert synergistic effects with 8-oxo-Guo to enhance inflammation. (A, B)** Protein expression changes in NLR, TLR, and downstream signaling pathways **(A)** and concentrations of TNF-α, MIP-2, IL-1β, and MCP-1 in culture supernatant **(B)** of MH-S cells after 4-h co-stimulation with 8-oxo-Guo and microRNAs that contained varying numbers of uridine residues. ∗*p* < 0.05, ∗∗∗*p* < 0.001. **(C, D)** Protein expression changes in relevant signaling pathways **(C)** and cytokine concentrations in the culture supernatant **(D)** of MH-S cells following 4-h stimulation with triple-uridine-containing mmu-miR-23a-3p or mmu-miR-10a-5p, either alone or combined with 8-oxo-Guo. ∗∗∗*p* < 0.001. **(E, F)** Heatmap of changes in plasma cytokine levels in mice after an injection with 8-oxo-Guo and miR-23a-3p. **(G)** Schematic diagram of the procedure for detecting inflammatory cytokines in mouse lung tissue. **(H)** Changes in inflammation-related cytokine levels in lung tissues in mice that were injected with 8-oxo-Guo and miR-23a-3p. **(I)** Molecular docking models of 8-oxo-Guo with NOD2, NOD2 with miR-23a-3p, and NOD2-8-oxo–Guo complex with miR-23a-3p.

In the present study, we evaluated miRNAs with varying numbers of uridine residues in their sequences to determine whether any of them synergistically enhance inflammation in conjunction with 8-oxo-Guo. The screening process focused on variations in the number of uracil residues within the miRNA sequences. miRNAs that contained one to three consecutive or non-consecutive U residues, as well as those with a high number of U residues but lacking consecutive U sequences, were selected and subsequently combined with 8-oxo-Guo for cell stimulation experiments. The Western blot and ELISA analyses indicated that mmu-miR-23a-3p and mmu-miR-10a-5p, which contain three consecutive U residues, exhibited a synergistic inflammatory response to 8-oxo-Guo, which was similar to the effect of polyU. Conversely, miRNAs with other sequences did not exert a significant synergistic inflammatory effect, likely because of the presence of fewer consecutive U residues (Fig. 5A and B, Supplementary Fig. 4A). Furthermore, mmu-miR-23a-3p and mmu-miR-10a-5p did not independently induce inflammation; rather, they potentiated the inflammatory response only in the presence of 8-oxo-Guo, with mmu-miR-23a-3p exerting a particularly pronounced effect (Fig. 5C and D, Supplementary Fig. 4B).

To assess the potential of miRNAs to synergistically stimulate the inflammatory response with 8-oxo-Guo *in vivo*, we co-injected mmu-miR-23a-3p and 8-oxo-Guo in the tail vein in mice. Four hours post-injection, mouse plasma was collected, and concentrations of 200 cytokines were measured using a cytokine microarray. The assays indicated that plasma levels of several proinflammatory cytokines significantly increased in mice that were treated with 8-oxo-Guo, including Activin A, Gremlin, IL-12p70, IL-1ra, IL-23, IL-28, IL-6, Resistin, secreted frizzled related protein 3, 6Ckine, B Lymphocyte Chemoattractant (BLC/CXCL13), C-C motif chemokine ligand 28 (CCL28), C-X-C motif chemokine ligand 16 (CXCL16), Eotaxin, Keratinocyte-derived Chemokine (KC), Lipopolysaccharide-Induced CXC Chemokine(LIX), MCP-1, MCP-5, macrophage-derived chemokine, MIP-1b, MIP-2, MIP-3a, Regulated Apon Activation, Normal T-cell Expressed and Secreted (RANTES), and stromal cell-derived factor 1α, compared with the control group (Fig. 5E). Moreover, the co-injection of 8-oxo-Guo and mmu-miR-23a-3p led to a further increase in levels of specific cytokines compared with 8-oxo-Guo alone, such as IL-12p40, Granzyme B, vascular endothelial growth factor receptor 1, CD36, CD30, pro-matrix metalloproteinase-9, A disintegrin and metalloproteinase with thrombospondin motifs 1, Dickkopf-1, signaling lymphocytic activation molecule, hepatocyte growth factor, thymus and activation-regulated chemokine, and Epigen (Fig. 5F). Gene Ontology (GO) enrichment analysis was conducted on cytokines that exhibited significant alterations in the co-stimulation groups that involved 8-oxo-Guo and mmu-miR-23a-3p. The analysis revealed that the differentially expressed cytokines were enriched in various biological processes, including cytokine-mediated signaling pathways, cell chemotaxis, monocyte migration, the regulation of immune effector processes, chemokine-mediated signaling pathways, cellular responses to lipopolysaccharides, responses to TNFs, cytokine production associated with immune responses, the positive regulation of immune effector processes, and the regulation of inflammatory responses (Supplementary Fig. 4C).

We next assessed inflammatory cytokine levels in mouse lung homogenates (Fig. 5G). The co-injection of 8-oxo-Guo and mmu-miR-23a-3p significantly elevated levels of TNF-α, IL-1β, IL-2, IL-10, granulocyte-macrophage colony-stimulating factor (GM-CSF), and interferon-γ (IFN-γ) compared with the control and notably increased IL-12p40, IL-10, and RANTES compared with 8-oxo-Guo alone (Fig. 5H). Building on our findings, the analysis of lung homogenates confirmed a robust synergistic inflammatory response between 8-oxo-Guo and mmu-miR-23a-3p *in vivo*. This provides direct evidence that the presence of a miRNA with a consecutive triple-uridine sequence enables 8-oxo-Guo to drive a more profound inflammatory response. This discovery extends the implications of our work, suggesting that the synergistic interaction between 8-oxo-Guo and U-rich miRNAs is a general mechanism that can exacerbate inflammation during viral infection and in non-infectious contexts.

To further elucidate the potential tripartite interaction between 8-oxo-Guo, NOD2, and mmu-miR-23a-3p, we conducted molecular docking simulations (Fig. 5I). We first docked 8-oxo-Guo to NOD2, revealing a binding energy of -8.7 kcal/mol. The interaction, stabilized by key residues (VAL-785, ASP-784, and ILE-236) via hydrogen bonds and hydrophobic forces, implies substantial affinity and potential functional relevance. We next examined the binding of NOD2 to mmu-miR-23a-3p, which showed a highly favorable binding energy (-383.15 kcal/mol; < -200) and high confidence score (0.9907; >0.7). The three-dimensional model depicted interactions with residues GLU-939, LYS-827, and ARG-857 and with hydrogen bonds that aided miRNA stabilization in the binding pocket. Importantly, when the preformed NOD2-8-oxo-Guo complex was docked with mmu-miR-23a-3p, it retained a high binding propensity (energy: -383.39 kcal/mol; confidence: 0.9907). The analysis of this ternary complex revealed conformational shifts. Interactions that involved residues GLU-939, ARG-857, and ASN-269 suggested that 8-oxo-Guo and the miRNA may bind synergistically or competitively within the NOD2 domain. Collectively, these computations demonstrate feasible binding between all three components and ligand-dependent alterations in NOD2's binding mode, structurally corroborating our experimental observations.

### 3.6 8-oxo-Guo-mediated suppression of alveolar epithelial cell growth via an inflammatory microenvironment in AMs

**Fig. 6:**
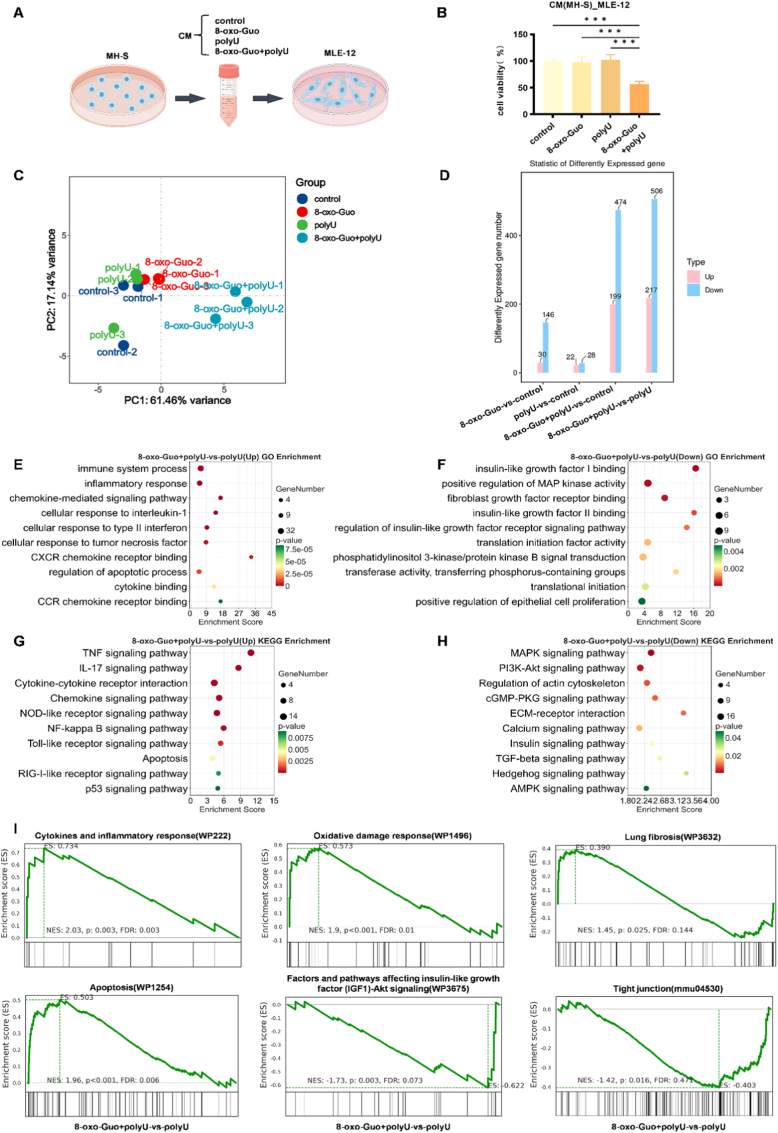
**8-oxo-Guo and polyU suppress alveolar epithelial cell growth through a macrophage-mediated inflammatory microenvironment. (A)** Schematic diagram of the MH-S and MLE-12 cell co-culture system. **(B)** Viability of MLE-12 cells after 24-h culture with CM that was derived from MH-S cells that were prestimulated for 24 h with 1 mM 8-oxo-Guo and 20 μg/ml polyU. ∗∗∗*p* < 0.001. **(C-I)** Transcriptome sequencing results of MLE-12 cells that were cultured for 24 h with CM from MH-S cells that were prestimulated for 24 h with 1 mM 8-oxo-Guo and 20 μg/ml polyU. **(C)** Principal Components Analysis plot. **(D)** Bar chart that compares the number of DEGs. **(E, F)** KEGG enrichment analysis of significantly up- and downregulated DEGs. **(G, H)** Gene Ontology enrichment analysis of significantly up- and downregulated DEGs. **(I)** GSEA of significantly up- and downregulated DEGs.

To assess the impact of 8-oxo-Guo-induced macrophage inflammation on alveolar epithelial cells, we established a co-culture system using MH-S and MLE-12 cells (Fig. 6A). Our results confirmed that the inflammatory microenvironment that was generated by 8-oxo-Guo and polyU suppressed MLE-12 cell survival. Conditioned medium (CM) from MH-S cells that were prestimulated with 8-oxo-Guo and polyU for 24 h significantly reduced MLE-12 viability compared with control CM, measured by the Cell Counting Kit-8 (CCK-8) assay (Fig. 6B).

The transcriptome sequencing of MLE-12 cells revealed distinct effects. Principal Components Analysis showed clear separation of the co-stimulation group from others on PC1 (61.46% variance), indicating a unique global transcriptomic profile (Fig. 6C). Consistent with this, the DEG analysis confirmed substantially more transcriptional changes with co-stimulation, with 723 DEGs versus polyU alone, far exceeding individual treatments (Fig. 6D).

Enrichment analyses (GO, KEGG, and GSEA) elucidated a convergent dual mechanism that suppressed proliferation. First, CM activated an inflammation-apoptosis network via innate immunity (e.g., TLR/NOD-like pathways), NF-κB-driven inflammation (TNF and IL-17 signaling), chemokine recruitment, p53 signaling, and apoptosis upregulation (Fig. 6E–G). Second, proliferative capacity was directly impaired through the downregulation of insulin-like growth factor binding/signaling, the inhibition of phosphoinositide 3-kinase–protein kinase B (PI3K-Akt) and MAPK pathways, and the blockade of translation initiation (Fig. 6F–H). The GSEA further revealed the activation of oxidative damage responses, DNA damage, cell cycle arrest, lung fibrosis pathways, and tight junction disruption, indicating epithelial barrier impairment and a pro-fibrotic microenvironment (Fig. 6I).

Overall, 8-oxo-Guo disrupts pulmonary homeostasis by creating a macrophage-mediated inflammatory microenvironment that drives alveolar epithelial cells into a damage-response state. This simultaneously activates proinflammatory/apoptotic pathways and represses regenerative growth signals, resulting in an “inflammatory-repair imbalance.”

## 4 Discussion and conclusion

The present study elucidated the significant role of 8-oxo-Guo in pulmonary inflammation and, for the first time, demonstrated its primary activation of the inflammatory signaling pathway via the NOD2 receptor. More importantly, the synergistic effect of 8-oxo-Guo and ssRNA intensified the inflammatory response, thereby facilitating the progression of inflammation. This discovery establishes a theoretical framework for elucidating the mechanism of 8-oxo-Guo in inflammation-related diseases.

The disruption of redox homeostasis causes oxidative damage to nucleic acids, lipids, and proteins. The aberrant accumulation of these damage products activates signaling cascades, upregulating proinflammatory cytokines and creating a positive-feedback inflammatory environment [51]. Oxidative stress activates NLRP3 and NF-κB, promoting the secretion of IL-1β and IL-18, linking oxidative stress, inflammation, and fibrosis [52]. Alveolar macrophages are central to initiating and progressing pulmonary fibrosis by regulating extracellular matrix deposition [53]. Although inflammation's role in idiopathic pulmonary fibrosis is debated, inflammatory mediators significantly drive fibrosis. Macrophages adapt phenotypically to environmental signals, participating in inflammation [54], whereas cytokines provoke pulmonary reactions and accelerate idiopathic pulmonary fibrosis pathogenesis [55]. In the present study, 8-oxo-Guo increased inflammatory cytokine levels in mice and MH-S cells, inducing the M1 polarization of AMs. As a unique nucleic acid oxidation marker, 8-oxo-Guo likely modulates macrophage polarization signaling networks, influencing proinflammatory/anti-inflammatory balance. Future research will investigate its chronic effects on pulmonary tissue, examining links to post-inflammatory fibrosis through collagen deposition and fibroblast proliferation and activation.

Notably, although 8-oxo-Guo did not directly elicit a significant inflammatory response in MLE-12 cells, inflammatory mediators that were secreted by MH-S cells inhibited MLE-12 cell proliferation. The interplay between pulmonary macrophages and alveolar epithelial cells is crucial in modulating inflammation. Oxidative stress activates inflammatory pathways via NF-κB. Cytokines, such as IL-1β and TNF-α, from macrophages amplify oxidative stress in adjacent endothelial and epithelial cells [56]. Under conditions like pulmonary infections or acute respiratory distress syndrome, compromised macrophages secrete proinflammatory cytokines (TNF-α, IL-1β, and MCP-1), recruiting inflammatory cells and impairing epithelial cell function and integrity [[57], [58], [59], [60]]. Furthermore, macrophages also secrete transforming growth factor-β1 (TGF-β1) to regulate epithelial proliferation and repair, controlling inflammation intensity and duration [61,62]. Dysregulation can lead to chronic inflammation and fibrosis [63]. The present study showed that 8-oxo-Guo exacerbates inflammation primarily through AMs as key effectors, while indirectly inhibiting alveolar epithelial cell proliferation.

The present study confirmed that 8-oxo-Guo significantly activates PRRs in pulmonary macrophages, including NOD2, TLR2, and NLRP3. Notably, NOD2 exhibited the most pronounced response to 8-oxo-Guo, potentially because of its rapid response characteristic as a cytosolic PRR. In alignment with research by Caruso et al. [64], NOD2 swiftly initiates the expression of proinflammatory cytokines, such as TNF-α and IL-8, via RIP2 kinase, thereby serving as a primary signaling catalyst for subsequent inflammatory pathways. The inhibitor experiments demonstrated that while the reactivity of TLR2 was less pronounced than NOD2, the inhibition of TLR2 could still attenuated certain 8-oxo-Guo-mediated inflammatory responses. This observation implies potential signal interference between NOD2 and TLR2, aligning with the “receptor complex model” that was proposed by Watanabe et al. [65]. According to this model, upon the recognition of bacterial lipoproteins by TLR2, myeloid differentiation primary response 88 (MyD88) is recruited, thereby enhancing stability of the NOD2-RIP2 signaling complex and establishing a positive feedback loop. Consequently, during initial stages of pulmonary infection, the rapid response that is initiated by NOD2 can effectively trigger a defensive mechanism. However, under prolonged stimulation, signal amplification that results from the synergistic interaction between TLR2 and NOD2 may exacerbate the inflammatory response. Compared to NOD2 and TLR2, activation of the NLRP3 inflammasome exerts a lesser impact on the intensity of the inflammatory response to 8-oxo-Guo. This suggests that NLRP3 activation is contingent on the preactivation of upstream PRRs, such as NOD2 or TLR2, which facilitate the NF-κB-mediated expression of NLRP3 and pro-IL-1β [66].

As a class of naturally occurring endogenous ssRNA molecules, miRNAs are involved in physiological and pathological processes and undergo alterations with aging [67]. The role of miRNAs in inflammation represents a burgeoning area of research, with specific miRNA sequences being pivotal in the regulation of inflammatory responses. Extracellular miRNAs have been identified as potent activators of innate immune responses, operating through TLR7 and MyD88-dependent pathways. This suggests that miRNAs with particular sequences may function as damage-associated molecular patterns to initiate inflammatory responses [68]. Certain miRNAs, particularly those that are rich in guanine-uracil (GU) sequences, can serve as ligands for TLRs, notably TLR7 and TLR8, thereby triggering proinflammatory signaling pathways [69]. Consequently, the interaction between miRNAs and cell membrane receptors is crucial in inflammation. The present study revealed that miRNAs with three consecutive uracils (e.g., mmu-miR-23a-3p and mmu-miR-10a-5p) synergistically amplify 8-oxo-Guo-induced inflammation via NLR and TLR pathways. The co-administration of mmu-miR-23a-3p and 8-oxo-Guo elevated proinflammatory cytokines in mouse plasma and lung tissue. Molecular docking indicated a strong NOD2-mmu-miR-23a-3p interaction, suggesting that such miRNAs collaborate with 8-oxo-Guo in infection-independent inflammatory processes.

Chronic inflammation is a hallmark of aging and intricately linked to numerous age-related diseases. Research indicates that chronic low-grade inflammation, termed “inflammaging,” progressively develops during aging and significantly influences pathophysiological mechanisms that underlie age-related diseases [70]. This chronic inflammatory condition impairs immune system function and is also associated with the onset and progression of various diseases, including metabolic disorders, cardiovascular diseases, and neurodegenerative diseases [71]. The biomarker 8-oxo-Guo indicates oxidative stress and nucleic acid damage. The present study elucidated its proinflammatory mechanism, expanding the oxidative stress-inflammation-disease framework and providing a theoretical basis for its role as an aging biomarker in chronic inflammation. Findings show that 8-oxo-Guo is a passive aging marker and key effector in “inflammaging.” In clinical practice, the dynamic monitoring of 8-oxo-Guo levels could enable the precise evaluation of therapeutic outcomes in aging-related inflammatory diseases and identify high-risk frail elderly patients. Ultimately, 8-oxo-Guo may shift from a diagnostic marker to a therapeutic target, supporting personalized anti-inflammatory treatments and advancing from assessing aging to actively delaying it.

Although the present study demonstrates an important role for 8-oxo-Guo in pulmonary inflammation through both *in vivo* and *in vitro* experiments, several limitations should be acknowledged. First, the *in vivo* experiments were performed using an acute exposure model with analysis conducted 4 h after tail vein injection of 8-oxo-Guo. This experimental setting primarily reflects early inflammatory responses and therefore may not fully recapitulate the persistent low-grade inflammation observed in chronic pulmonary disease settings. Thus, the long-term effects of 8-oxo-Guo under chronic inflammatory conditions remain to be further investigated. In addition, although our findings identify AMs as important responsive cells to 8-oxo-Guo, the pulmonary microenvironment involves complex interactions among multiple cell populations, including endothelial and mesenchymal cells, which may also contribute to or modulate 8-oxo-Guo-induced inflammation. Such multicellular interactions may not be fully captured in the current acute experimental model. Future studies using chronic exposure or sustained administration models will be important to further evaluate the pathological role of 8-oxo-Guo in chronic pulmonary inflammation.

In summary, the present findings elucidate the molecular mechanisms by which 8-oxo-Guo influences pulmonary inflammatory responses via PRRs. They highlight the novel central regulatory role of NOD2 and synergistic stimulation by 8-oxo-Guo with miRNAs that contain three consecutive uracils, markedly enhancing the secretion of lung inflammation-related factors. These findings expand the link between RNA oxidative damage and pulmonary inflammation, providing a theoretical foundation for understanding the pathogenesis of pulmonary inflammatory diseases. The 8-oxo-Guo–NOD2 axis is emerging as a potential therapeutic target. Critically, the present study deepens insights into the regulatory network of aging-related chronic inflammation and lays a molecular foundation for developing aging assessment systems based on nucleoside oxide markers.

## Funding

This work was supported by the 10.13039/501100012166Chinese Academy of Medical Sciences Innovation Fund for Medical Sciences (2021-I2M-1-050); the National Key R&D Program of China (grant no. 2024YFA1109102); the 10.13039/501100001809National Natural Science Foundation of China (82304565); and National High Level Hospital Clinical Research Funding (BJ-2024-138 and BJ-2023-246).

## CRediT authorship contribution statement

**Rui Li:** Formal analysis, Investigation, Methodology, Validation, Visualization, Writing – original draft. **Jia-Xin Pan:** Investigation, Validation. **Gang Sheng:** Investigation, Validation. **Xu-Fan Gao:** Investigation, Validation. **Ran Huan:** Investigation, Validation. **Ruo-Mei Qi:** Methodology, Supervision, Writing – review & editing. **Jin Li:** Methodology, Project administration, Supervision, Writing – review & editing. **Jian-Ping Cai:** Conceptualization, Methodology, Resources, Supervision, Writing – review & editing.

## Declaration of competing interest

The authors declare that they have no known competing financial interests or personal relationships that could have appeared to influence the work reported in this paper.

## Data Availability

All data supporting the findings of this study are available within the article and its supplementary materials or from the corresponding author upon reasonable request.
