# Supplementary material for: 8-oxo-7,8-dihydroguanosine (8-oxo-Guo) drives pulmonary inflammatory pathways through pattern recognition receptors
Source: Redox Biol. 2026 May 14;94:104211. doi: 10.1016/j.redox.2026.104211 (PMC13223851; doi:10.1016/j.redox.2026.104211)
Supplement: Multimedia component 1 [file mmc1.docx]

**Supplementary Information**

**Supplementary Fig. 1**


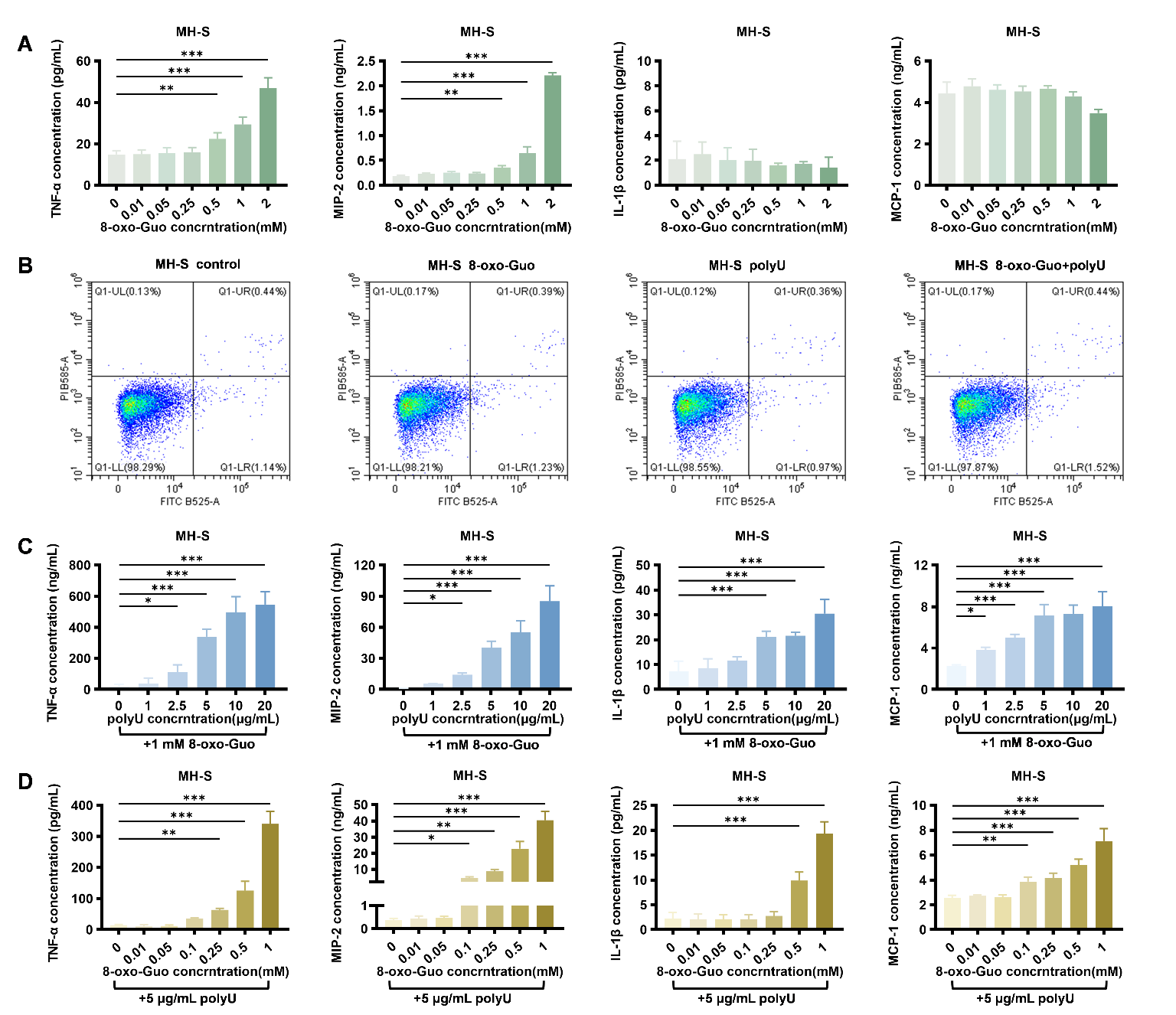


**Supplementary Figure 1. 8-oxo-Guo and polyU concentration-dependently increase inflammatory cytokines. (A)**Concentrations of TNF-α, MIP-2, IL-1β, and MCP-1 in the culture supernatant of MH-S cells that were stimulated with varying concentrations of 8-oxo-Guo. ***p* < 0.01, ****p* < 0.001. **(B)**Apoptosis of MH-S cells after 24-h stimulation with 1 mM 8-oxo-Guo and 20 μg/ml polyU. **(C)**Concentrations of TNF-α, MIP-2, IL-1β, and MCP-1 in the culture supernatant of MH-S cells following co-stimulation with 1 mM 8-oxo-Guo and varying concentrations of polyU. **p* < 0.05, ****p* < 0.001. **(D)**Concentrations of TNF-α, MIP-2, IL-1β, and MCP-1 in the culture supernatant of MH-S cells following co-stimulation with 5 μg/ml polyU and varying concentrations of 8-oxo-Guo. **p* < 0.05, ***p* < 0.01, ****p* < 0.001.

**Supplementary Fig. 2**

**
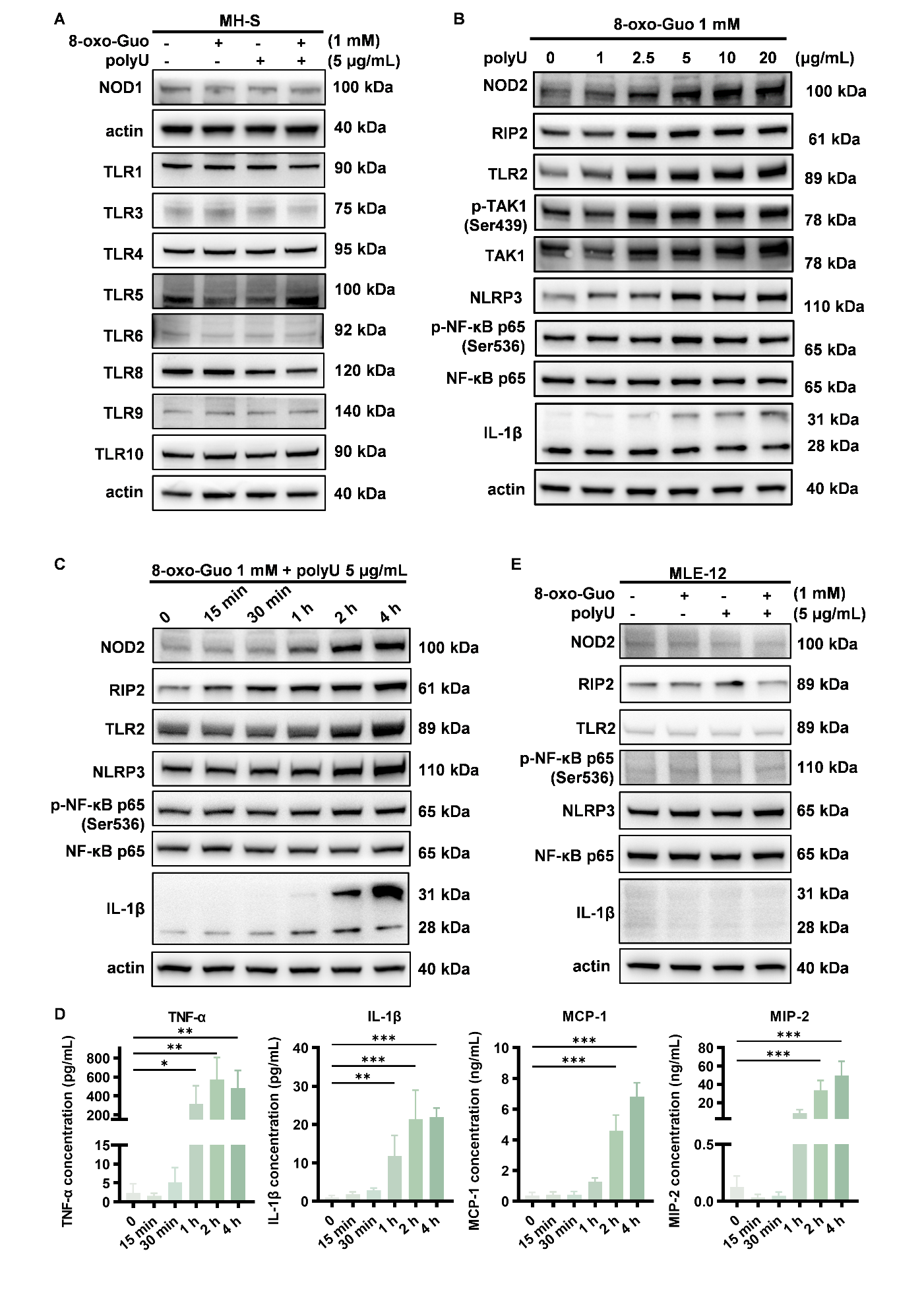
**

**Supplementary Figure 2. 8-oxo-Guo and polyU stimulate inflammation via NLR and TLR signaling pathways. (A)**Protein expression levels of NOD1, TLR1, TLR3, TLR4, TLR5, TLR6, TLR8, TLR9, and TLR10 in MH-S cells after 4-h stimulation with 8-oxo-Guo alone or combined with polyU. **(B)** Changes in the expression of proteins that are associated with NLR, TLR, and downstream signaling pathways in MH-S cells after 4-h co-stimulation with 1 mM 8-oxo-Guo and varying concentrations of polyU. **(C, D)**Time-dependent changes in the expression of proteins in relevant signaling pathways **(C)** and concentrations of TNF-α, MIP-2, IL-1β, and MCP-1 in the culture supernatant **(D)** following co-stimulation with 8-oxo-Guo and polyU. **p* < 0.05, ***p* < 0.01, ****p* < 0.001. **(E)** Changes in the expression of NLR, TLR, and downstream IL-1β proteins in MLE-12 cells after 4-h co-stimulation with 8-oxo-Guo and polyU.


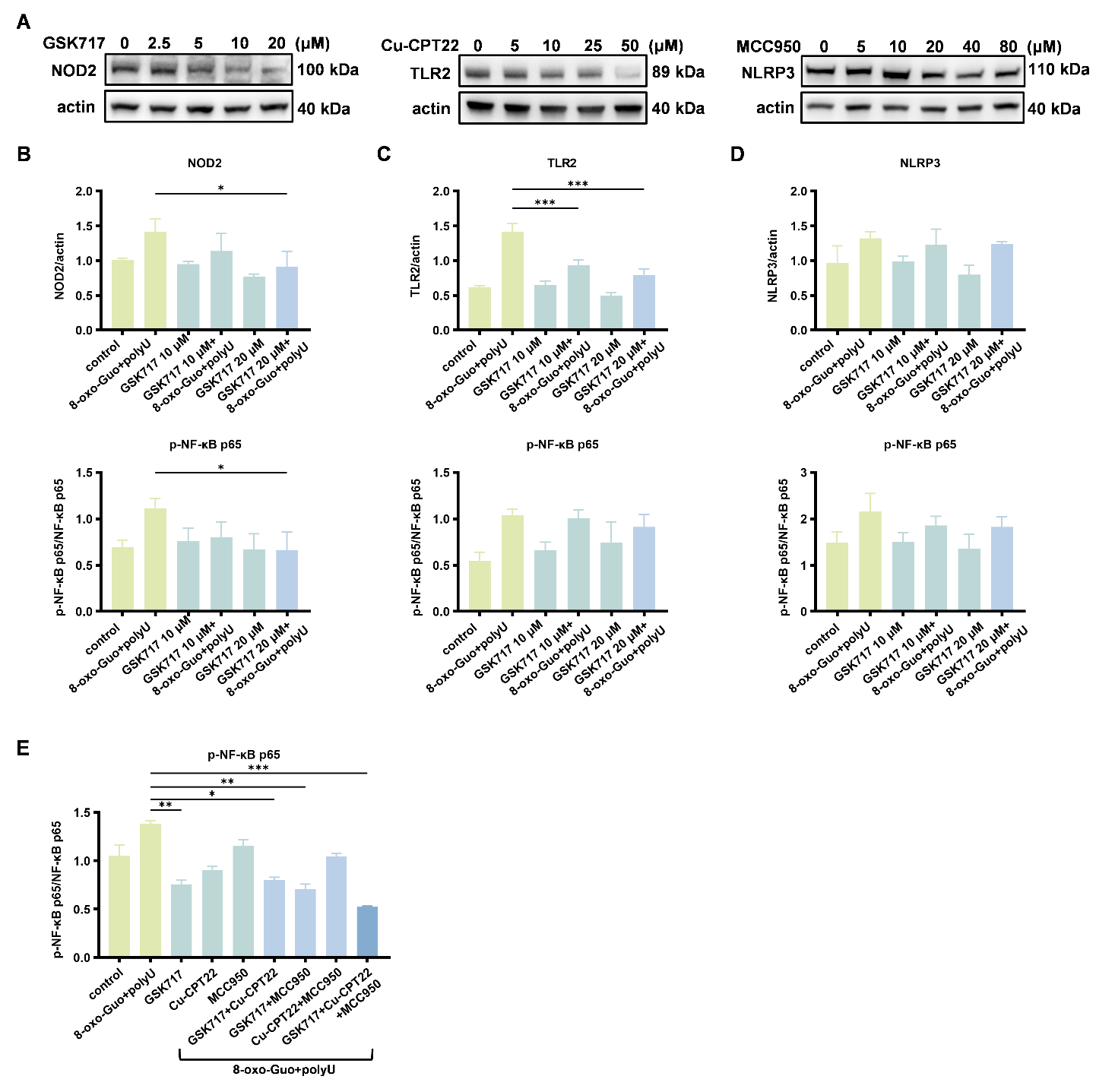
**Supplementary Fig. 3**

**Supplementary Figure 3. GSK717, Cu-CPT22, and MCC950 suppress the expression of NOD2, TLR2, and NLRP3 proteins. (A)**Expression levels of NOD2, TLR2, and NLRP3 proteins following treatment with varying concentrations of GSK717, Cu-CPT22, and MCC950. **(B)**Grayscale quantification of NOD2 and p-NF-κB p65 protein expression following treatment with different concentrations of GSK717. **p* < 0.05. **(C)**Grayscale quantification of TLR2 and p-NF-κB p65 protein expression following treatment with different concentrations of Cu-CPT22. ****p* < 0.001. **(D)**Grayscale quantification of NLRP3 and p-NF-κB p65 protein expression following treatment with different concentrations of MCC950. **(E)**Grayscale quantification of p-NF-κB p65 protein expression following combined treatment with the three inhibitors. **p* < 0.05, ***p* < 0.01, ****p* < 0.001.


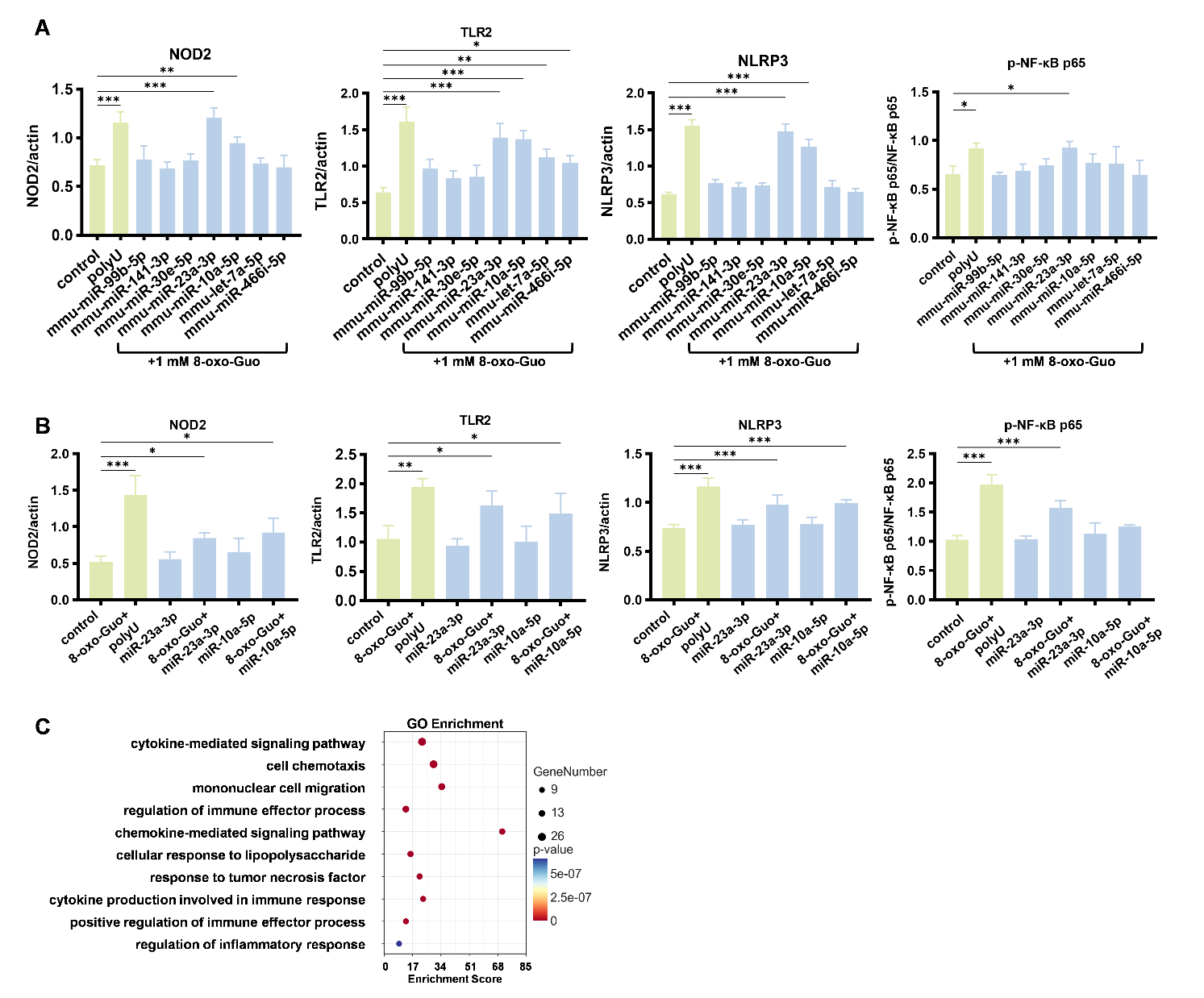
**Supplementary Fig. 4**

**Supplementary Figure 4. MicroRNAs that contain three consecutive uridines also synergistically enhance inflammation with 8-oxo-Guo. (A)** Grayscale quantification of NLR, TLR, and p-NF-κB p65 protein expression in MH-S cells after 4-h co-stimulation with 8-oxo-Guo and microRNAs that contained varying numbers of uridine residues. **p* < 0.05, ***p* < 0.01, ****p* < 0.001. **(B)** Grayscale quantification of NLR, TLR, and p-NF-κB p65 protein expression in MH-S cells following 4-h stimulation with triple-uridine-containing mmu-miR-23a-3p or mmu-miR-10a-5p, either alone or combined with 8-oxo-Guo. **p* < 0.05, ***p* < 0.01, ****p* < 0.001. **(C)** Enriched GO terms of biological processes for cytokines that were significantly upregulated in mouse plasma following an injection with 8-oxo-Guo and miR-23a-3p.

**Supplementary Tables**

**Supplementary Table 1.** Primary antibodies for Western blot.

| **Primary antibody** | **Dilution ratio** | **Catalog no.** | **Manufacturer** |
| --- | --- | --- | --- |
| NOD2 | 1:250 | PA5-104317 | Invitrogen |
| RIP2 | 1:1000 | R30192 | Zenbio |
| p-TAK1 (Ser439) | 1:1000 | R25849 | Zenbio |
| TAK1 | 1:1000 | 12330-2-Ap | Proteintech |
| NLRP3  Cleaved-caspase 1  Caspase 1  IL-1β  TLR2  TLR7  p-IκBα (Ser32/36) | 1:1000  1:1000  1:1000  1:1000  1:1000  1:500  1:1000 | 15101  341030  24232  ab283818  ab209217  sc-57403  10268-1-AP | Cell Signaling Technology  Zenbio  Cell Signaling Technology  Abcam  Abcam  Santa Cruz Biotechnology  Proteintech |
| IκBα  p-NF-κB p65 (Ser536)  NF-κB p65  iNOS  COX2  p-SAPK/JNK (Thr183/Tyr185)  SAPK/JNK  p-p38 MAPK (Thr180/Tyr185)  p38 MAPK | 1:1000  1:1000  1:1000  1:1000  1:1000  1:1000  1:1000  1:1000  1:1000 | 10268-1-AP  3033  8242  340668  12282  4668  9252  4631  9212 | Proteintech  Cell Signaling Technology  Cell Signaling Technology  Zenbio  Cell Signaling Technology  Cell Signaling Technology  Cell Signaling Technology  Cell Signaling Technology  Cell Signaling Technology |
| p-p44/42 MAPK (Erk1/2) (Thr202/Tyr204)  p44/42MAPK (Erk1/2)  p-JAK2 (Tyr1007/1008)  JAK2  p-STAT3 (Ser727)  STAT3  Vinculin  β-actin  NOD1  TLR1  TLR3  TLR4  TLR5  TLR6  TLR8  TLR9  TLR10 | 1:1000  1:1000  1:1000  1:1000  1:1000  1:1000  1:500  1:1000  1:1000  1:500  1:1000  1:500  1:1000  1:1000  1:500  1:500  1:500 | 4370  4695  3771  3230  94994  9139  sc-73614  4970  32256  sc-514399  ab307442  sc-293072  66570-1-Ig  22240-1-Ap  sc-373760  sc-515921  sc-293300 | Cell Signaling Technology  Cell Signaling Technology  Cell Signaling Technology  Cell Signaling Technology  Cell Signaling Technology  Cell Signaling Technology  Santa Cruz Biotechnology  Cell Signaling Technology  SAB  Santa Cruz Biotechnology  Abcam  Santa Cruz Biotechnology  Proteintech  Proteintech  Santa Cruz Biotechnology  Santa Cruz Biotechnology  Santa Cruz Biotechnology |

**Supplementary Table 2.** Sequences of ssRNA.

| **ssRNA** | **Sequences (5’-3’)** |
| --- | --- |
| polyU | UsUsUsUsUsUsUsUsUsUsUsUsUsUsUsUsUsUsU |
| *mmu-miR-99b-5p*  *mmu-miR-141-3p*  *mmu-miR-30e-5p*  *mmu-miR-23a-3p*  *mmu-miR-10a-5p*  *mmu-let-7a-5p*  *mmu-miR-466i-5p* | CACCCGUAGAACCGACCUUGCG  UAACACUGUCUGGUAAAGAUGG  UGUAAACAUCCUUGACUGGAAG  AUCACAUUGCCAGGGAUUUCC  UACCCUGUAGAUCCGAAUUUGUG  UGAGGUAGUAGGUUGUAUAGUU  UGUGUGUGUGUGUGUGUGUG |

**Supplementary Table 3.** Sequences of PCR primers.

| **Species** | **qPCR Primer** | **Sequences (5’-3’)** |
| --- | --- | --- |
| Mouse | *NOD2* Forward | CAGGTCTCCGAGAGGGTACTG |
| Mouse | *NOD2* Reverse | GCTACGGATGAGCCAAATGAAG |
| Mouse | *TLR2* Forward | TCTAAAGTCGATCCGCGACAT |
| Mouse | *TLR2* Reverse | CTACGGGCAGTGGTGAAAACT |
| Mouse | *NLRP3* Forward | ATTACCCGCCCGAGAAAGG |
| Mouse | *NLRP3* Reverse | TCGCAGCAAAGATCCACACAG |
| Mouse | *TNF-α* Forward | CCTGCAGCTGGAGAGTGTGGAT |
| Mouse | *TNF-α* Reverse | TGTGCTCTGCTTGTGAGGTGCT |
| Mouse | *MIP-2* Forward | CCAACCACCAGGCTACAGG |
| Mouse | *MIP-2* Reverse | GCGTCACACTCAAGCTCTG |
| Mouse | *IL-1β* Forward | CCTGCAGCTGGAGAGTGTGGAT |
| Mouse | *IL-1β* Reverse | TGTGCTCTGCTTGTGAGGTGCT |
| Mouse | *MCP-1* Forward | CCTGCTGCTACTCATTCACCA |
| Mouse | *MCP-1* Reverse | ATTCCTTCTTGGGGTCAGCA |
| Mouse | *GAPDH* Forward | CCTCTCCAGAACATCATCC |
| Mouse | *GAPDH* Reverse | GTGTCGCTGTTGAAGTCAG |
